# Harnessing the power of physicochemical material property screening to direct breast epithelial and breast cancer cells

**DOI:** 10.1016/j.bioactmat.2025.04.003

**Published:** 2025-04-26

**Authors:** Lisa E. Tromp, Rik de Jong, Torben A.B. van der Boon, Alejandro Reina Mahecha, Ruud Bank, Jan de Boer, Patrick van Rijn

**Affiliations:** a University of Groningen, University Medical Center Groningen, Department of Biomaterials and Biomedical Technology FB-40, A. Deusinglaan 1, 9713 AV, Groningen, the Netherlands; b University of Groningen, University Medical Center Groningen, Department of Pathology and Medical Biology, A. Deusinglaan 1, 9713 AV, Groningen, the Netherlands; c Eindhoven University of Technology, Department of Biomedical Engineering, Institute for Complex Molecular Systems, 5612 AZ, Eindhoven, the Netherlands

**Keywords:** High-throughput screening, Biointerfaces, Breast implants

## Abstract

Understanding cell-material interactions is crucial for advancing biomedical applications, influencing cellular behavior and medical device performance. Material properties can be manipulated to direct cell responses, benefiting applications from regenerative medicine to implantable devices such as silicone breast implants. Knowledge about the interaction differences between healthy and cancer cells with implants may guide implant design to more precisely influence cell adhesion and proliferation of healthy cells while inhibiting cancer cells, tailoring outcomes to specific cellular responses. To show-case this potential, breast epithelial cells and breast cancer cells were investigated regarding their interaction with a broad range of combined physicochemical properties. This study employed a silicone-based high-throughput screening method utilizing Double Orthogonal Gradients (DOGs) to investigate the influence of topography, stiffness, and wettability on breast epithelial cells (MCF10a) and breast cancer cells (MCF7). Results show distinct cellular responses, including decreased proliferation rates in both MCF10a and MCF7 cells with the introduction of surface topography and the dominant influence of wettability on cell adhesion, proliferation, and cluster formation. The screening identified specific regions of interest (ROIs) where MCF10a cell proliferation outperformed MCF7 cells and that topography inhibits cluster formation (tumorigenesis), offering potential prospects for the creation of novel implant surfaces.

## Highlights

• MCF10a and MCF7 cells be selectively triggered in their behavior by combinations of wettability, surface wrinkled topography, and stiffness
• Addition of surface topography decreases MCF10a and MCF7 cell proliferation rates and inhibits MCF7 spheroid formation
• Wettability outperforms topography and stiffness concerning cell adhesion and proliferation of MCF10a and MCF7 cells
• From a wide range of combinations wettability, surface wrinkled topography, and stiffness, there is only a select number of combinations proliferation of MCF10a cells is enhanced and that of MCF7 cells inhibited

## Graphical abstract

**Figure:**
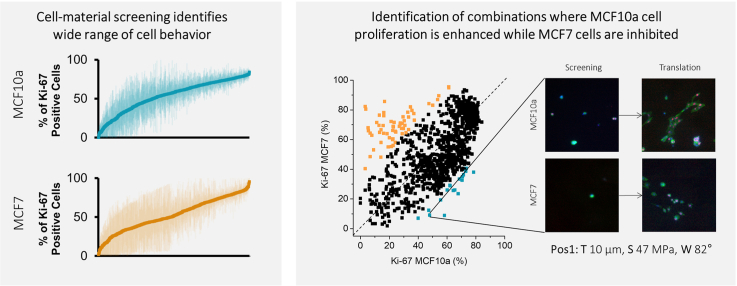

## 1 Introduction

Understanding cell-material interactions is crucial for advancing biomedical applications, as these interactions can significantly influence cellular behavior, and, consequently, the performance of medical devices [1,2]. Material properties influence biological outcomes, and they can be strategically manipulated to direct the cell response towards favorable effects [[3], [4], [5]]. This principle holds for a wide range of applications, from regenerative medicine and tissue engineering to more fundamental (stem) cell research [[6], [7], [8], [9], [10]]. Similar strategies are employed in developing implantable devices [11,12]. For instance, silicone breast implants, used both in reconstructive and cosmetic surgery, rely heavily on biocompatibility of their materials to ensure safe and predictable outcomes [13]. In the evolution of breast implant design, surface texturing became a well-known method to improve implant stability and reduce rates of capsular contracture [14,15]. Currently, various methods altering the surface topography in terms of roughness or other forms of topography are applied to breast implants available on the market and are shown to influence implant performance [16,17]. For instance, Langer et al. demonstrated how surface topography of silicone breast implants affects the foreign body response [12,18]. Moreover, other implant surface modifications such as surface chemistry or mechanical properties are extensively being studied to improve cellular responses to the biomaterial [[18], [19], [20]].

However, breast implant safety remains an important topic due to emerging complications. Recently, evidence was found for an increased incidence of a rare peripheral T-cell lymphoma, Breast Implant Associated Anaplastic Large Cell Lymphoma (BIA-ALCL), in patients with textured breast implants [[21], [22], [23]]. Moreover, even if all the breast cancer is removed during mastectomy, there remains a potential for the cancer to return [24]. The literature concerning breast cancer recurrence after breast implants is contradictory, leaving gaps in our understanding of this matter [25]. Although some studies suggest no relation between implant use and recurrence [26,27], another study found a higher risk of breast cancer recurrence with the use of textured surface implants compared to smooth surface implants [28]. However, despite a possible link between inflammation and breast cancer recurrence, there is limited understanding of how cancer cells are influenced by various material properties, especially also in relationship to healthy cells [28,29].

In general, studies fall behind in the understanding of the interactions between different cell types and silicone materials following the implantation in the human body. While most in vitro or in vivo studies focus on the reaction of macrophages or fibroblasts towards silicone gel or silicone elastomer, few studies have explored the interactions of a wider range of cell types, including breast epithelial or even cancer cells and silicone gel or surfaces [30,31]. As cell-material interactions cannot be reliably generalized across different cell types, such as healthy and cancerous cells, it is important to identify their different behavior to different material surfaces separately. These insights can guide implant design to more precisely influence cell adhesion and proliferation of healthy cells while inhibiting cancer cells, tailoring outcomes to specific cellular responses.

However, traditional research methods still have limitations, as they often focus on only one altered parameter with very specific chosen values, leading to incomplete information on both the biological and materials aspects of the interface. In order to be able to define an optimum desired outcome, high-throughput screening methodologies that increase their data output within one experimental setup can rapidly increase knowledge [32,33]. Therefore, in this study, the Biomaterial Advanced Cell Screening (BiomACS) was utilized, a high-throughput screening approach, using breast epithelial cells (MCF10a) and breast cancer cells (MCF7) to elucidate their differences in cell-material interactions. The MCF10a cell line is widely used as a model for normal human mammary epithelial cells, while MCF7 cells are used as a model for breast cancer due to their display of several features of transformed mammary epithelium [34,35]. The objective of this research was to investigate how specific combinations of surface wrinkled topography, stiffness, and wettability on polydimethylsiloxane (PDMS) substrates affect the adhesion and proliferation of healthy (MCF10a) and cancerous (MCF7) breast cells. We aimed to identify regions of interest (ROIs) where these material properties selectively promote or inhibit cell responses, offering insights for potential breast implant design.

The cell-material screening is performed on the recently developed BiomACS platform based on Double Orthogonal Gradients (DOG), on which every position on the surface has a unique combination of surface wrinkled topography, stiffness, and wettability to which cells can respond [20,36]. The DOGs are created by a set of sequential air plasma oxidation treatments on PDMS substrates under a 90-degree angle to each other. With this platform, the cell response to an extremely broad range of surface parameter combinations can be studied. This screening method allows the identification of ROIs revealing optimum cell behavior. Hence, different behavior between both cell types in terms of cell adhesion and proliferation can reveal specific combinations of material properties that promote MCF10a cells while inhibiting MCF7 cancer cells.

To perform the screening, MCF10a breast epithelial cells and MCF7 breast cancer cells were seeded onto the DOG substrates and cultured for 24 h and 72 h, after which the cells were fixated. Cells were stained for nucleus, cytoskeleton, and proliferation marker Ki-67, imaged, and the cell density, cell area, Ki-67 positive cells, and cancer cell aggregate formation were quantified. Scatter plots, representing almost 1000 unique surfaces, were prepared that show the different trends between the two cell types. ROIs with specific material combinations were identified where MCF7 cell proliferation is diminished compared to MCF10a cell proliferations, which might offer the potential to implement these findings in new breast implant designs. Additionally, MCF7 cancer cell clusters, that can be seen as small and simplified models of tumors, replicating some aspects of the tumor microenvironment [37] were also shown to be influenced by the underlying physicochemical surface properties and could be exploited in novel designs for future implants.

## 2 Results

### 2.1 Cell screening shows diverse behavior of cells to material properties

The screening platform, as previously reported [20], consisting of four different DOG samples Stiffness-Wettability (**S-W**), Topography-Stiffness (**T-S**), Topography-Wettability (**T-W**), and Topography-Stiffness|Wettability (**T-S|W**), was prepared, characterized, and used for cell screening experiments (Figure S1-2). When referring to the topography gradient, consisting of an aligned wrinkled topography with increasing wavelength (1–10 μm) and amplitude (100–1000 nm), its respective wavelength will be discussed, but the corresponding amplitude can be found in Fig. S1.

**Fig. 1:**
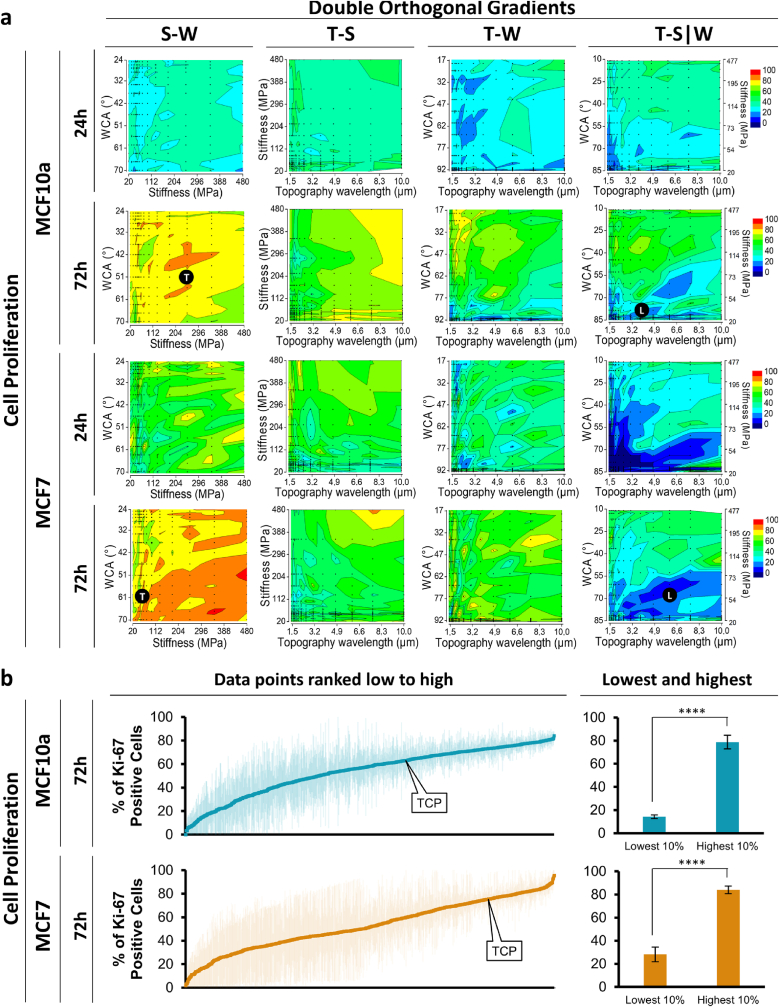
**DOG screening overview. a**, Screening overview showing the heatmaps (average of n = 3) of the cell proliferation at 24 h and 72 h for both the MCF10a and MCF7 cells quantified as Ki-67 positive nuclei. The heatmaps corresponding to the cell density and cell area can be found in the supplementary figures. **b**, Scatter plots showing all data points from one screening (4 DOGs) ranked from low to high. Tissue culture polystyrene (TCP) was used as a control. The 10 % lowest and 10 % highest scoring datapoints are shown on the side and are shown to be significantly different (p < 0.0001). Annotation of black dot with “T” or “L” corresponds to the representative fluorescence images of “Top” and “Low” hits as shown in Fig. 2.

MCF10a breast epithelial cells and MCF7 breast cancer cells were seeded on the DOG samples and cultured for 24 h and 72 h, followed by fixation and immunostaining. The entire DOG area was imaged using a TissueFAXs automated fluorescence microscope (Fig. S3). Cell density (nuclei count mm^−2^), cell area (phalloidin area μm^2^ per cell), and cell proliferation (percentage of Ki-67 positive nuclei count) were quantified and shown in readily interpretable heatmaps that effectively visualize the effect of the different gradients on the biological parameters. Fig. 1a shows the heatmaps of the cell proliferation for the MCF10a and MCF7 side by side. These heatmaps provide an overview of the four different DOGs, each consisting of a topography, stiffness, or wettability gradient on each axis, while the color scale represents the biological behavior (cell proliferation, % Ki-67 positive nuclei) of the cells on each specific spot of the gradients. From these heatmaps, it is shown that cells represent different cell behavior depending on the specific combination of material properties present on specific locations on the DOGs. In general, proliferation levels at 72 h are higher than at 24 h. At 72 h, more material property-specific behavior is observed as proliferation rates of both >80 % and <30 % can be found in different locations of the heatmaps. More specifically, cells residing on the more hydrophobic side (WCA >60°) of the **T-S|W** DOG, both MCF10a and MCF7 cells, show lower proliferation rates (<30 % Ki-67 positive) compared to cells residing on the more hydrophilic side (WCA <40°, >50 % Ki-67 positive). Moreover, a combined parameter effect can be seen by looking into specific areas of the heatmaps. For example, for MCF10a cells after 72 h on the **T-S|W** DOG, it appears that wettability and stiffness are the most dominant factors while alteration of topography does not impact the proliferation as much, especially on the more hydrophilic side of the DOG of WCA <40°. However, within the WCA range of around 70°, a topography of 3 μm shows higher proliferation (∼50 %) compared to a topography of 6 μm (∼20 %). Thus, by observing the cell behavior on each axis, the heatmaps provide insights into the specific combinations in which secondary or tertiary parameters can impact the cell response.

**Fig. 2:**
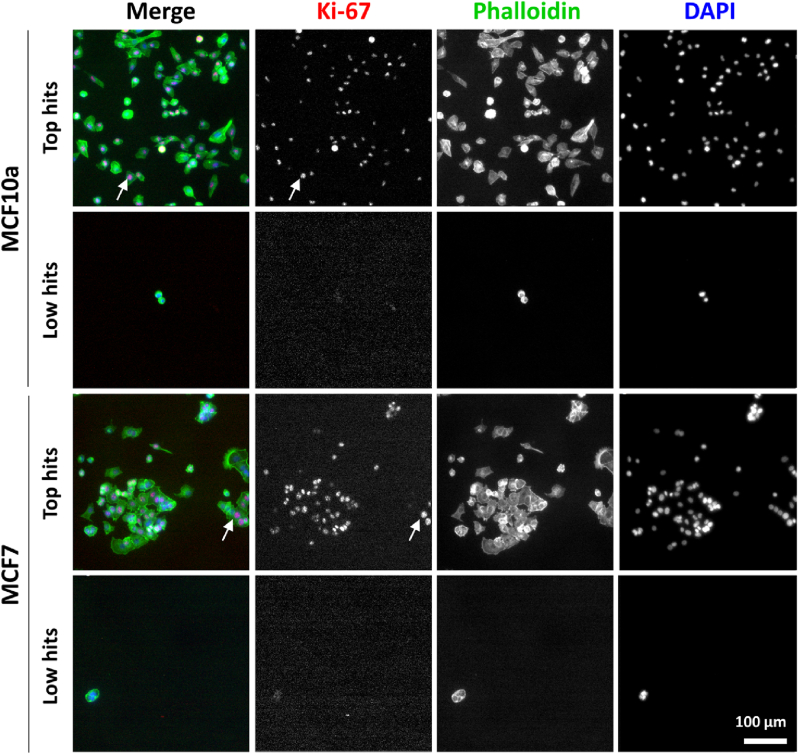
**Top- and low-hit Ki-67-expressing proliferative cells after 72 h of cell culture**. Cells were stained for Ki-67 (red), phalloidin (green), and nuclei (blue). Scale bar is 100 μm. White arrows show examples of Ki-67 positive nuclei. The underlying material properties of each image are: MCF10a top hit: S-W 51° 251 MPa, MCF10a low hit: T-S|W 3.8 μm 82° 47 MPa, MCF7 top hit: S-W 63° 42 MPa, MCF7 low hit: T-SW 6 μm 68° 63 MPa.

The quantified cell behavior on each position on all four DOGs, representing a specific combination of physicochemical material properties, is regarded as a single datapoint (small black dots in Fig. 1a) and totaled to a combined dataset per cell type and time point. In order to visualize the wide range of cell behavior towards the different material properties, all datapoints were sorted from low to high according to their cell density, cell area, and cell proliferation. These scatterplots, both for MCF10a and MCF7 proliferation are shown in Fig. 1b, and representative fluorescence images of top- and low-hit cells are shown in Fig. 2, and have been annotated in the heatmaps of Fig. 1a. From the fluorescence images, it can be clearly seen that top-hits have many Ki-67 positive nuclei, as well as higher cell densities and larger cell area, compared to the low-hits. Moreover, it can be seen that MCF10a cells are more dispersed, while MCF7 cells are more in clusters, which will be discussed more in detail later.

It is evident from the scatterplots that varying combinations of physicochemical material properties can lead to distinct cell behaviors with a significant increase in Ki-67 % between the 10 % lowest (<10 % Ki-67 positive) and 10 % highest (>80 % Ki-67 positive) hits. The cell doubling time for these highest and lowest hits was calculated based on the cell density values at 72 and 24 h. For the MCF7 cells, the 10 % most proliferative hits have an average doubling time of 20.2 ± 8.6 h, while the 10 % least proliferative hits have an average doubling time of 40.3 ± 122.7 h. For the MCF10 cells, the highest 10 % correspond to an average doubling time of 22.6 ± 34.5 h, and the lowest 10 % of −73.3 ± 204.1 h. The negative doubling time indicates that there was a decrease in cell number between 72h and 24h, corresponding with no proliferation, migration, or cell death. In general, both the highest proliferating cells, both the MCF10a and MCF7 cells, show lower cell doubling times, as expected for cells that are actively dividing. The least proliferative cells have higher cell doubling times, or even negative values, with a high standard deviation.

All other heatmaps and scatter plots of cell proliferation, cell density, and cell area are found in Figs. S4–6.

### 2.2 Material properties can stimulate MCF10a and inhibit MCF7 cells

**Fig. 3:**
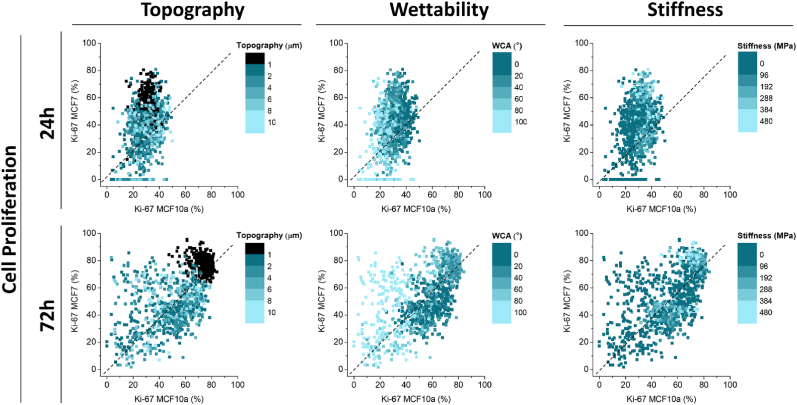
**Scatter plots comparing the effect of each individual physicochemical parameter of the DOG between MCF10a and MCF7 cells.** The material parameters (topography, wettability, and stiffness on the same data points, shown with the corresponding color scale) were shown on the biological output (cell proliferation, % Ki-67 positive cells) for the MCF10a vs. the MCF7 cells. Similar plots showing the scatter plots for cell density and cell area can be found in Fig. S7.

To determine specific combinations of physicochemical material properties that repel malignant cells but enhance healthy cell adhesion and function, the results from the screening were compared between the two cell types. In order to effectively compare the differences in cell behavior between the two cell types, scatter plots were made that plot each data point, representing a specific combination of material properties as present on the DOG samples, according to the biological outcome (cell proliferation, cell density, and cell area) for both the MCF10a (x-axis) and MCF7 (y-axis) cells (Fig. 3, Fig. S7). The datapoints’ topography, wettability, and stiffness values are visualized using different color scales on the same scatterplot. In this way, the effect of the individual material properties, irrespective of which DOG sample they originate from, can be observed and trends can be determined. Moreover, specific combinations of material properties can be identified that have a stimulating effect on the MCF10a cells while showing an inhibiting effect on the MCF7 cells, potentially opening the doors for materials that selectively inhibit cancer cells.

Generally, the initial 24-h period shows that MCF10a cells display a lower proliferation rate and size, and spread and proliferate more after 72 h (Fig. S7 – **Cell Area**). Contrarily, MCF7 cells display a wide range of proliferative behavior and cell area after already 24 h. These observations suggest that MCF10a cells require some time to acclimate to the biomaterial surface.

When looking at the effect of the individual parameters; topography, wettability, and stiffness, multiple trends are observed from the scatter plots. Of note, cell proliferation at 72 h for both the MCF7 and MCF10a was significantly higher on the flat **S-W** DOG compared to the **T-S**, **T-W**, and **T-S|W** DOGs (73 ± 6 % vs. 47 ± 18 % for MCF10a, 78 ± 6 % vs. 44 ± 16 % for MCF7, Fig. S8), suggesting that addition of topography leads to a reduced proliferation.

Likewise, a decrease in cell area is seen for both cell types after 72 h on the surfaces with topography compared to the flat surfaces (Fig. S7 – **Cell Area &; 72h)**. However, apart from the distinction between flat surfaces and the introduction of winkled topography, no impact on the specific size of the wrinkled topography was observed. Flat surfaces show higher proliferation rates compared to surfaces with wrinkled topography, but further varying the wrinkle size does not result in a clear trend with proliferation. For wettability, the scatterplots indicate a trend towards increased proliferation on more hydrophilic surfaces for MCF10a cells after both 24 h and 72 h, but this trend is not observed for the MCF7 cells. This trend corresponds well with the cell density and cell area of MCF10a cells after 72 h (Fig. S7). For the MCF7 cells, only a trend in cell area is observed, where cells tend to spread more on hydrophilic surfaces. This suggests a stronger effect of wettability on MCF10a cells compared to MCF7 cells. As for stiffness, a minor trend can be observed between surfaces with a high stiffness and a high proliferation rate.

**Fig. 4:**
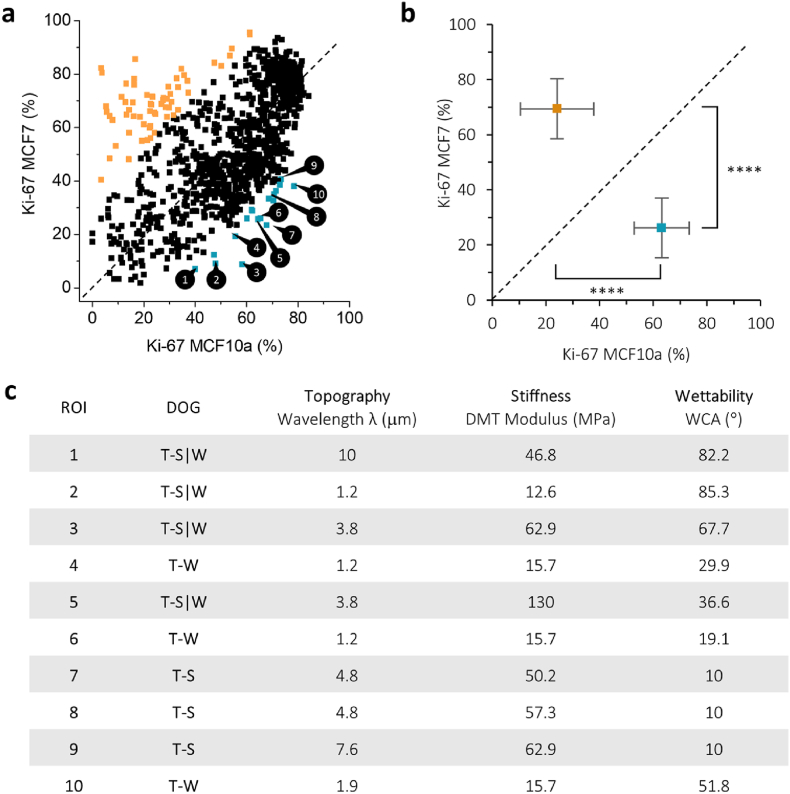
**ROIs were chosen that lead to a higher proliferation after 72 h for MCF10a than MCF7 cells. a**, Regions of interest (ROIs) are chosen as points furthest away from the dashed line. In orange and blue, points are indicated that show an individual statistical difference (P < 0.05) when comparing the proliferation percentage of MCF7 vs. MCF10a. Orange or blue dots indicate an increase for either MCF7 or MCF10a proliferation, respectively. **b**, Points from **a** were grouped and show a significant different in both MCF10a and MCF7 Ki-67 positive cells percentage (p < 0.0001). **c**, Individual DOG values for each ROI chosen in **a** are shown. Corresponding fluorescence images for each ROI can be found in Fig. S9.

Regions of interest (ROIs) were identified at which the proliferation of MCF10a cells was higher than the MCF7 cells, indicating a potential combination of material properties that would enhance healthy cell proliferation while reducing cancer cell proliferation, as shown in Fig. 4. These combinations of material properties were chosen as the points furthest away from the diagonal (dashed line) shown in Fig. 3. Fig. S9 shows the corresponding fluorescence cell images on those specific combinations of material properties.

**Fig. 5:**
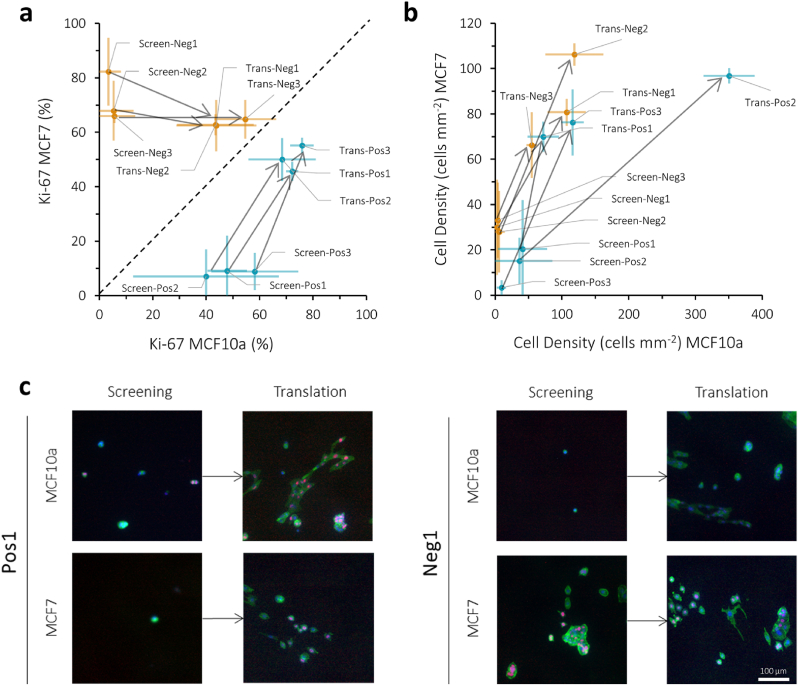
ROIs were chosen from the screening as “positive” when MCF10a Ki-67 % > MCF7 Ki-67 % (labelled as Pos1, Pos2, Pos3, shown in blue) and “negative” when MCF7 Ki-67 % > MCF10a Ki-67 % (labelled as Neg1, Neg2, Neg3, shown in orange). **a**, Cell experiments were repeated on translational (labelled as Trans) substrates with those specific material properties and compared to the proliferation (% Ki-67 positive cells after 72 h of cell culture) rates from the screening (Screen). **b**, Cell density after 72 h was compared between the screening and translation findings. **c**, Fluorescence images of two example ROIs, Pos1 and Neg1. Cells were stained for Ki-67 (red), phalloidin (green), and DAPI (blue). Corresponding fluorescence images for the other ROIs can be found in Fig. S10. The scale bar is 100 μm.

Three ROIs were chosen that indicate higher proliferation for MCF10a than MCF7 cells in the screening, to be labelled as “positive”: Pos1, Pos2, and Pos3 (Fig. 5). Contrarily, three ROIs were chosen that indicate higher proliferation for MCF7 than MCF10a cells, labelled “negative”, Neg1, Neg2, and Neg3. The specific combination of material properties characterizing these ROIs (Table 2) were translated to uniform samples. Treatment parameters were identified to generate homogeneous substrates bearing those desired properties (Figure S10), which were then utilized in repeated translational cell experiments to compare against the original screening outcomes. Fig. 5a shows that the translational cell experiments reveal an apparent shift in proliferation. Pos1, Pos2, and Pos3 all exhibit higher proliferation rates for both MCF10a and MCF7 cells in the translational experiments compared to the original screening findings. However, these points all remain on the correct side of the dashed diagonal line, indicating that the trend of higher MCF10a proliferation compared to MCF7 remains, although the effect is smaller. Similarly, for conditions Neg1, Neg2, and Neg3, a comparable trend is observed with higher MCF10a proliferation in the translational experiments compared to the screening. This discrepancy may be attributed to differences in cell density between the screening and translational experiments, as shown in Fig. 5b–c and fluorescence images in Figure S11. All conditions from the translational experiments displayed an increased cell density compared to the screening.

**Fig. 6:**
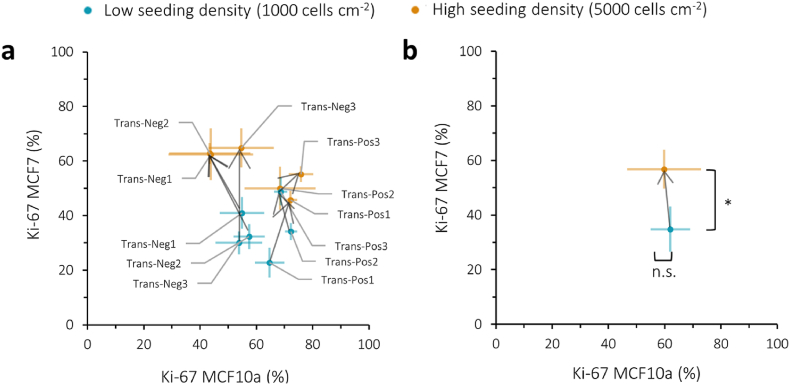
Proliferation quantified as % Ki-67 positive cells after 72 h for both MCF7 and MCF10a cells compared between the translational cell experiments on translated ROIs for both low (shown in blue, 1000 cells cm^−2^) and high (shown in orange, 5000 cells cm^−2^) seeding density. **a**, Individual values for each ROI compared between low and high seeding density. **b**, All low and high seeding density datapoints from **a** were grouped and compared for statistical difference (n.s.: p > 0.05, ∗: p < 0.05).

To investigate whether the observed higher proliferation rates in the translational experiments were indeed influenced by increased cell density, the same experiment was repeated on the translated ROIs using a lower initial seeding density (Fig. 6, Figure S12), specifically 1000 cells cm^−2^ compared to the original 5000 cells cm^−2^. Upon grouping all ROIs according to low or high seeding density (Fig. 6b), it seems that cell density affects cell behavior in a different manner for MCF7 and MCF10a cells. At lower seeding density, MCF7 cells exhibit less Ki-67 positive cells compared to the experiments with higher seeding density, whereas MCF10a cells display similar numbers of Ki-67 positive cells between the low and high seeding density experiments. It therefore appears that MCF10a cells outperform MCF7 cells, causing all ROIs, whether labelled as “negative” or “positive” to shift to the lower right side of the graph in Fig. 6a.

### 2.3 Quantitative analysis of the effect of wettability, topography, and stiffness on cell behavior

**Fig. 7:**
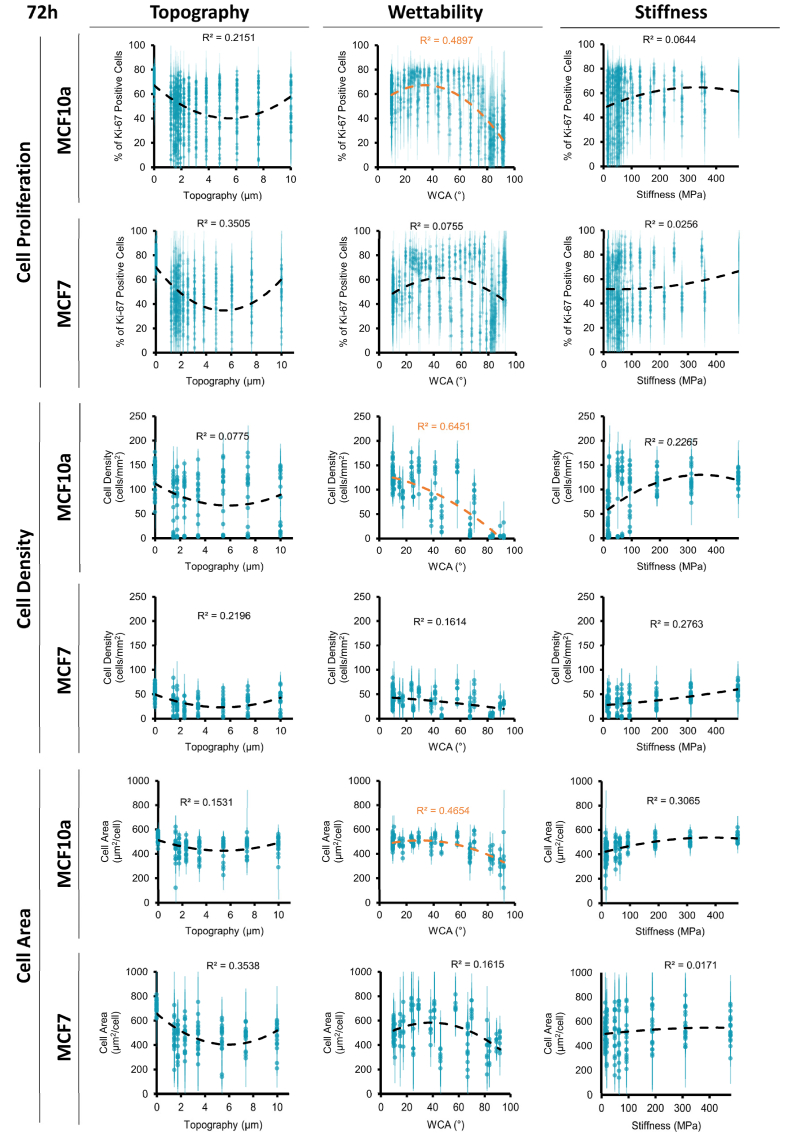
**Nonlinear regression analyses of individual material properties after 72 h of cell culture.** Data points were plotted with the value for topography, wettability, or stiffness against cell proliferation, cell density, and cell area and nonlinear second-order polynomial regression was performed. Trend lines with the highest R^2^ values are shown on each graph, with values higher than 0.4 deemed as representative, shown in orange.

Additionally, to develop quantitative relationships between the different material properties and biological outcome, the properties were correlated to the cell proliferation, cell density, and cell area, using nonlinear regression (Fig. 7 for 72 h, Fig. S13 for 24 h). WCA is moderately correlated with increased cell proliferation, higher cell density, and larger cell area in MCF10a cells (R^2^ = 0.49, 0.65, and 0.47, respectively), with an increase found while reducing the WCA from 100° to around 50°, settling at an optimum proliferation found at a WCA of 30–40° (Fig. 7) after which proliferation reaches a plateau. Although, a similar trend can be observed for MCF7 cells after 72 h, with an optimum around 40–60°, no significant correlation was found. However, for the latter cell type, at 24 h, WCA is moderately correlated with cell density and cell area (R^2^ = 0.57 and 0.61, respectively, Fig. S13) This outcome suggests that MCF10a cells, after 72 h, are more affected by changes in surface wettability compared to MCF7, which is also observed from the scatter plots (Fig. 3). In general, this wettability effect seems to be the most dominant factor in provoking high cell adhesion and proliferation. However, specific combinations with topography and stiffness also show higher proliferation of MCF10a cells at higher or lower wettability, in combination with a lower MCF7 cell proliferation, as indicated in Fig. 4. This showcases the importance of the combinatorial effect of multiple surface parameters on cell response.

The low correlation values for both topography and stiffness might be reasoned by the dominant influence of wettability. However, despite their relatively low R^2^ values, these plots illustrate a trend where increased stiffness values align with higher cell density and cell area. In the case of topography, there is indeed a distinction observed between the flat surfaces (indicated as 0 μm) and surfaces with wrinkled topography (ranging from 1 to 10 μm). However, no clear correlations were identified within the range of the different topographical features.

Besides these nonlinear regression analyses, a partial least squares (PLS) analysis was performed. Fig. S14 shows the contributions of each variable relative to the others, separately per DOG. The data on the R^2^ values and number of components are shown in Fig. S15 This allows to assess whether specific trends are dependent on the other parameters they are combined with. Although the R^2^ values are low, the variable importance shows a higher importance of wettability for MCF10a cells compared to MCF7 cells, for which the importance is only high on the **T-S|W** DOG. Topography only seems to have a high variable importance when combined with stiffness, both for the MCF10a and MCF7 cells.

### 2.4 MCF7 early cluster formation is inhibited on surfaces with topography

**Fig. 8:**
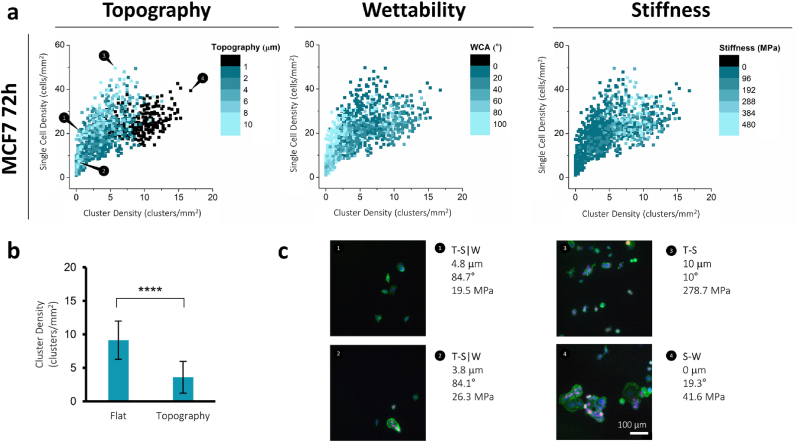
**Cluster density of MCF7 cells after 72 h of cell culture.** Cluster density is quantified as every individual phalloidin area in contact larger than 2000 μm^2^ mm^−2^, corresponding to two or more cells. Single cell density is quantified as every individual phalloidin area in contact smaller than 2000 μm^2^ mm^−2^, corresponding to single cells. **a**, Cluster density is shown against single cell density, with each data point representing a combination of physicochemical properties that is shown with its according color scale. **b**, All datapoints from the flat S-W DOG are grouped and show a statistically significant difference (p < 0.0001) with all datapoints from the DOGs containing topography (T-S|W, T-S, T-W). **c**, Fluorescent images are shown for the numbered regions with phalloidin (green), Ki-67 (red), and DAPI (blue). Scale bar is 200 μm.

One of the striking features of breast cancer cells is to develop clusters of cells, tumors, which is represented here as cluster formation by the MCF7 cells, which was not observed for the healthy MCF10a cells. During the screening experiments, cluster formation occurred, as already visible in Fig. 2 for the top-hits of MCF7 cells, and the early indication of the formation of clusters of MCF7 cells occurred after 72 h, which was not seen for the MCF10a cells. This formation of three-dimensional clusters can occur under certain conditions, and may be seen as small and simplified models of tumors [37]. From the original screening data, the early formation of such clusters by MCF7 cells was quantified along with single cell density to analyze if there is a relationship between the different material properties and the formation of these clusters compared to the individual cells present at the material surface (Fig. 8, Figure S16). In general, most material properties show a presence of both single cells and small clusters. Moreover, an increase of cluster density appears to be correlated to an increase in average cluster area (Figure S17a). What stands out is that the highest cluster density is found on the flat surface (Fig. 8a – **Topography**, black dots), which is in line with the findings for cell proliferation. Therefore, it appears that the presence of topography allows the cells to attach more individually to the material surface, while a flat surface steers the cells to attach more to each other, forming clusters. This is further confirmed by grouping all datapoints on flat surfaces and all datapoints on surfaces with topography, which shows a significantly increased cluster density on flat surfaces (9.1 ± 2.9 clusters mm^−2^) compared to topography surfaces (3.6 ± 2.4 clusters mm^−2^) (Fig. 8b, Figure S17b).

## 3 Discussion

In this study, a high-throughput system was utilized to elucidate the different cell-material interactions of breast epithelial cells and breast cancer cells towards a wide range of combined physicochemical material properties. The innovative DOG platform facilitated the investigation of a vast number of cell-material interactions towards different surface wrinkled topography, stiffness, and wettability. From the screening, specific combinations were identified that show a higher proliferation of the breast epithelial cells compared to the breast cancer cells.

Within our cell screening, wettability was generally the most dominant factor for predicting cell adhesion and proliferation, although specific combinations of all three parameters (wettability, topography, and stiffness) can induce a differential cell response. From the literature, it is known that surface wettability controls adhesion of cellular proteins and thus has an effect on cell behavior [38]. Our results are consistent with previous studies, where modification of PDMS with plasma treatment improved wettability, leading to enhanced cell adhesion and growth [20,[39], [40], [41]]. Silicone breast implants used in the clinic exhibit hydrophobic surfaces with contact angles ranging between 108° and 142°, but it remains the question whether this is optimal for cell responses [42]. Alterations in wettability can alter protein adhesion, and surfaces with intermediate wettability (not strongly hydrophilic or hydrophobic) are shown to induce appropriate distribution, orientation, and conformation of proteins on the surface to allow cell attachment [18,43]. For example, a moderate contact angle of 70° was found optimal for in vitro culture of fibroblasts [44]. However, each tested condition used an entirely different material, making it challenging to attribute cell adhesion solely to wettability, since the different materials differ in surface chemistry and stiffness. In a study using breast cells, MCF10a cells were grown on PDMS-based substrates coated with polyethylene-glycol (PEG), poly(lactide-co-glycolic acid) (PLGA), and sodium alginate (SA), leading to reduced surface WCAs and increased cell growth [45]. In our study, we were able to screen an extremely wide range of different WCAs on the same material, while decoupling the effect of topography and stiffness. The findings indicate that for MCF10a cells, increased wettability leads to an increased cell density, with WCA of 30–40° being optimal for cell proliferation after 72 h of culture. Contrarily, MCF7 cells showed enhanced cell adhesion to increased wettability after 24 h, but this effect is not visible anymore in the cell density after 72 h, suggesting that wettability primarily influences early cell adhesion. MCF10a may rely more heavily on specific adhesion-mediated signals, such as different protein adsorption to different hydrophobicity, meanwhile MCF7 are less sensitive to these cues. After 72 h, formation of MCF7 cell aggregates was observed, which could indicate stronger cell-cell adhesions overshadowing the cell-material interactions and reducing the impact of wettability. This effect of aggregation was not seen in the MCF10a cells, perhaps indicating that cell-material interactions were stronger than cell-cell interactions.

An intriguing finding from the screening was the influence of topography on cell proliferation. Specifically, the results show higher cell proliferation rates for both the MCF10a and MCF7 on the flat **S-W** DOG, compared to all the DOGs containing topography (**T-S**, **T-W**, **T-S|W**) that show a wide distribution of proliferation rates ranging from low to high. This suggests that the introduction of aligned wrinkled topographical features might limit proliferation. This effect partially aligns with the findings of Lim et al. who investigated the impact of microgratings on cell behavior and found a diminished proliferation in non-cancer breast epithelial cells (MCF10a) on 3 μm microgratings [46]. This effect could be explained by inducing an optimal dimension for the cell surface receptors to sense in such a way that leads to reorganization of the cellular cytoskeleton and subsequent reduction in proliferation [47]. Notably, the study by Lim et al. found no reduction in malignant breast cancer cells (MDA-MB-231 and MCF7) on the microgratings, which is contradicting our findings in which MCF7 cells do have higher proliferation rates on the flat **S-W** DOG compared to all DOGs containing topography. This discrepancy can be accounted for by the effect of the other material parameters stiffness and wettability. These altered material properties might be able to overrule the ability of MCF7 cells to adapt to the topographical features and maintain high proliferation capacity. This result shows the importance and the impact of studying the effect of combined material properties compared to studies that vary only one parameter. Besides proliferation, a decrease of cell area on surfaces with topography was seen compared to the flat surfaces. The incorporation of topography might hinder complete cell spreading, confining the cells to some extent. Anisotropic topographical features in the form of grooves and wrinkles have an effect on cell adhesion and spreading, and can guide cells in a certain direction by contact guidance [48,49].

A minor trend was seen between surfaces with high stiffness and high proliferation rate, despite their low R^2^ values. This is in line with literature that states that better cell anchorage at stiffer surfaces can lead to increased cell spreading [50]. However, it should be noted that this effect could be caused by the wettability effect the **T-S|W** DOG, in which the both parameters of stiffness and wettability are interlinked. As the individual effect of each parameter can be altered in combination with the other parameters, it is important to keep in mind that each point represents a combination of the three parameters leading to the biological outcome. While the DOG methodology is designed to systematically explore combined effects, these parameters are not entirely independent. For example, stiffness can affect deformation of the surface topography under cell forces, or topography can impact the wettability of a surface. Topography on a hydrophobic surface (WCA >90°) can trap air underneath the topography on which a water droplets rests, increasing its apparent hydrophobicity. Contrarily, on a hydrophilic surface (WCA <90°), a water droplet can adapt to the surface topography, resulting in a lower contact angle [51]. However, this effect is caused by the presence of a gas phase during the measurements, which is absent in a completely wetted system such as cell culture. During the characterization stage of the gradients, the WCA was therefore validated on flat samples to assess surface wetting based on surface polarity (chemistry), and exclude any variations induced by topography. These topography-related wettability changes could have an impact on cell behavior, although it is expected that the mechano-sensing of the cells towards topography plays a more dominant role. The results of this screening should be interpreted with an understanding of the parameter overlaps, focusing on their combined effect. Specifically, the identification of combinations of material properties and their outcome on cell behavior, the main focus of the screening, is not directly affected by interdependence of variables. The specific combinations of topography, stiffness, and wettability can be used to finetune the desired cell behavior and enable the identification of an optimum, one of the strengths of the current technology.

While the current research focused only on aligned wrinkled topography, stiffness, and wettability, it is important to note other parameters that could influence cells, thus limiting this study. We chose to describe our current surface topography by its respective wavelength and amplitude, as this most directly relates to the type of topography sensed by the cells. However, other types of introducing topography might differentially affect cell behavior. For example, surface roughness, often quantified with Ra measurements, can be introduced in a wide range of designs, varying from smooth (Ra <10 μm), microtextured (10 μm < Ra <50 μm), to macrotextured (Ra >50 μm), and clearly has an effect on the FBR to an implant [12]. The manufacturing process, such as salt-loss techniques or negative contact imprinting, affects further roughness parameters such as pore density, diameter, skewness, and kurtosis [52]. These can have different impact on cell behavior, allowing for different attachment points, tissue ingrowth, and cell differentiation [18,30,53]. In general, smooth breast implant surfaces have been associated with higher rates of capsular contracture compared to textured versions. Smooth surfaces limit the sites for tissue ingrowth and reduce tissue adherence, while textured implants allow a more multidirectional orientation of collagen fibers, inhibiting the contractive properties of the capsule formed around the implant. Moreover, other types of surface structuring such as micropatterns [54,55], biomimetic surfaces [16], and predefined combinations of features [56], have been explored. Tailoring the adhesive areas that cells can attach to can be used to instruct cell phenotype and function, such as differentiation, proliferation, and motility. For example, a specific design of 4 × 2 μm micropatterns has been shown to have an anti-fibrotic effect [54]. Combined, our findings on the addition of aligned wrinkles cannot directly be translated to other types of surface topography. Besides this, factors such as surface free energy, surface chemistry, and charge could also play a role in what makes an optimal interface with tissues and cells [38,57,58]. Hence, the properties investigated in this study could limit the current findings.

The effect of material properties on this MCF7 cluster formation was further analyzed, as this might be seen as a simplified tumor model. MCF7 cells might be more prone to early cluster formation compared to MCF10a, which is a non-tumorigenic line. This difference may be due to the intrinsic properties of MCF7 cells and their behavior in cancer progression [59]. Interestingly, surface topography seems to determine whether cells remain isolated or cluster into clusters. The topography might guide cell attachment by favoring individual cell adhesion to the substrate, while flat surfaces seem to support intercellular adhesion and increase cluster formation. Investigating cadherin-mediated cell-cell interactions and associated signaling pathways could provide more insights into the mechanisms behind this aggregation and further tumor progression [60]. Topographical features, in the form of microchannels, have shown before to enable cancer cells to attach as single cells to high-curvature channel bottoms [61], which might be comparable to the grooves present in our topography system displaying similar results. Other researchers have shown the potential of MCF7 cells to form larger, 3D clusters, which could be of use as in vitro tumor models [59,62]. The prevention of such cluster formation, at least in the early stage, could be an indication of reduced tumorigenesis and would therefore be of interest for longer time period studies that aim to reduce tumor formation. Furthermore, currently only a 2d measurement could be made to investigate cluster size, but in the future z-stack imaging should be included to demonstrate the 3D organization and potential further growth from early clusters into structures similar to spheroids. Extending the culture period would provide insights into the temporal changes of how these spheroids progress into potential tumors.

From the translational experiments, where combinations of materials properties from specific ROIs of the screening were translated onto homogeneous substrates for comparative cell studies, discrepancies with the original screening findings were found. In the case of the “negative” ROIs, where MCF7 proliferation initially outperformed that of MCF10a cells, the translational experiments revealed a shift towards higher proliferation of MCF10a cells. Contrarily, for the “positive” ROIs, MCF7 proliferation increased on the translated substrates. The possible complexity that gradients bring to the experiments might not be easily compared and translated towards homogeneous substrates. Within the screening, it is possible that cells migrate towards regions with preferential material properties, resulting in some regions remaining less populated, thereby explaining the lower cell density observed. On the homogeneous substrates of the translational experiments, this cue to migrate is diminished without the presence of a gradient, resulting in higher cell density and possibly higher proliferation rates. Therefore, by aiming to reduce the initial seeding density, it was possible to better match the cell density as seen at 72 h during the screening and investigate the effect on the proliferation rates. By conducting the same translational experiment with two different seeding densities, we observed that varying cell density can indeed impact cell proliferation. This phenomenon has been previously observed [63], revealing that as initial cell density increases, cancer cells require less time to adapt to their environment, thereby stimulating proliferation. However, it is worth noting that cell density can also inhibit proliferation in highly confluent environments due to contact inhibition for noncancerous cells [64]. In our studies, seeding densities were utilized that would still result in single cells to avoid such effects of complete confluency, as the restricting area dimensions could have an effect on cell proliferation. Furthermore, by switching to larger, homogeneous substrates as done in the ROI translational experiments, it was possible to assess a larger surface area. Combined, cell density, whether high or low, emerges as an important biological factor influencing cell behavior, which cannot be fully elucidated with the screening platform used in this study alone. Future studies should incorporate additional biological factors, such as cell density, as well as the influence of cytokines and growth factors on the respective cell behavior towards specific materials. This approach will give a more comprehensive understanding of how various biological cues interact with material properties to modulate cell behavior.

While the primary focus of this study centers on breast cells, both normal and cancerous, it is worth noting that this research approach can be applied towards various cell types. Our screening method enables the efficient screening of a wide range of material properties, facilitating the identification of specific combinations that can give the implant surface a therapeutic character. In the past, this screening approach has first been explored only using mesenchymal stem cells (MSCs) in order to validate the system and test for initial cell-adhesion [20]. Furthermore, the system has been applied to biomaterial-associated fibrosis [65], which is caused by an excessive foreign body response (FBR) to the implant material [66]. As it is clear that the material properties of the implant influence the FBR [12], alternative strategies can be developed that leverage the material's surface to actively modulate cell responses. By screening how fibroblasts or macrophages respond to different material properties, it could be possible to prevent their differentiation into myofibroblasts or polarization into pro-inflammatory macrophages through modification of the implant's surface [67]. In a recent study, the differentiation of fibroblasts to myofibroblasts was screened on the DOG system, and specific combinations were identified that result in high or low-fibrotic cell behavior [65]. Placing the earlier findings in perspective with the current study, the specific ROIs identified have been different for each specific application, and combinations that have resulted in beneficial results for preventing fibrosis do not necessarily match with the ROIs that enhance MCF10a cells while inhibiting MCF7 cells. Moreover, the role of topography in suppressing cancer cluster formation is a novel finding and was not seen before using other cell types. Therefore, when deciding specific material properties for implant design, it is important to individually screen for each specific complication.

The high-throughput screening platform used in this study is a valuable tool for pinpointing specific material properties associated with distinct cell behaviors, but its implications into biomaterial design require additional investigation. The concept of designing a cancer-inhibiting implant surface could be compared to the “race for the surface” phenomenon often described in the development of biomaterial-associated infection [68]. During the race for the surface, the dynamic competition that takes place between host cells and bacteria for colonization of the implant surface is a predictor for its medical success or failure [69,70]. This phenomenon could take place similarly in the context of cancer, describing the interactions between cancer cells and normal tissue cells with respect to their competition for space and resources within the body. Breast cancer cells can use the implant surface as a niche for survival, growth, and metastasis, depending on its microenvironment [71,72]. The material properties can enhance adhesion, proliferation, and invasion of cancer cells, but also protect them from immune surveillance. On the other hand, normal host cells such as breast epithelial cells can form a protective layer on the implant surface and potentially prevent the adhesion and invasion of breast cancer cells. Therefore, a side-by-side comparison of the cell-material interactions of the different cell types could lead to “hits” that can steer the implant interactions to a favorable outcome. By applying the hypothesis of the “race for the surface” phenomenon, we anticipate that cancer recurrence can be inhibited through the stimulation of healthy cells while preventing cancer cells. ROIs were identified where MCF10a cell proliferation was enhanced while MCF7 cell proliferation was reduced, which could hold promise for the development of breast implant surfaces that prevent cancer growth while stimulating proliferation of healthy tissue. However, it remains unclear whether induction of such cell behavior would indeed contribute to the prevention of cancer recurrence in the presence of a breast implant. Therefore, finding a true optimum of material properties remains a challenge, as it is currently unknown which cellular behavior leads to a specific outcome in the clinic. Translational research would be needed that apply these findings to more large-scale and complex situations taking into account more biological parameters.

Further research is needed to elucidate the underlying mechanisms driving these effects, by focusing on pathway analyses to determine how specific signaling cascades mediate cellular adhesion and proliferation in response to surface properties. One promising area of investigation is the role of integrins, critical mediators of cell-material interactions [73,74]. Moreover, key signaling pathways such as the FAK (Focal Adhesion Kinase) and ERK (Extracellular signal-Regulated Kinase) cascade should be explored, as they regulate cell adhesion, migration, and can respond to material cues [75]. Moreover, this study only investigated the individual cell-material interactions, but real physiological conditions are more complex, where multiple cells at once are interaction with both the material, other cells, and surrounding tissue. Co-culture studies with different ratios of MCF10a to MCF7 cells would provide deeper insights into the dynamic interactions between healthy and malignant cells. This enables to test whether the proposed material properties effectively inhibit cancer cells while promoting the healthy cells. However, predicting the relevant ratio of MCF10a to MCF7 would be extremely challenging, and this would greatly affect the findings. Therefore, cell-based experiments alone may not fully capture the complexity of the in vivo environment. Ultimately, in vivo experiments are needed to validate the findings of this study for potential implant applications using the material properties of the found ROIs. Animal models will aid to assess the efficacy and long-term performance of these specific materials in a more complex setting, including tissue integration, immune response, cytokine signaling, and overall therapeutic potential. In such complicated models, more complications can occur simultaneously, and it might be a challenge to identify an implant material that can solve multiple problems at once.

Concluding, this study highlights the importance of screening multiple physicochemical surface parameters in a combined fashion to uncover the intricate relationships between different types of cells and materials.

## 4 Methods

### 4.1 DOG formation and characterization

**Fig. 9:**
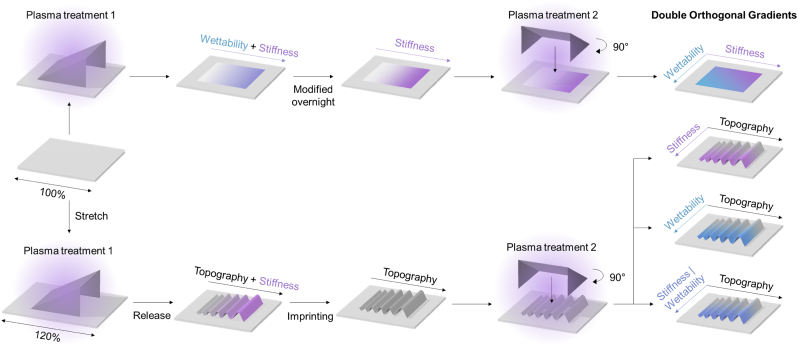
Schematic overview of the double orthogonal gradient (DOG) generation. The gradients are based on sequential shielded plasma treatments perpendicular to each other, combining both topography, stiffness, and/or wettability.

The DOG screening platform consists of four different substrates that have two orthogonally oriented surface parameter gradients, with the third parameter remaining unaltered (Fig. 9). The three surface parameter gradients of topography (**T**), stiffness (**S**), and wettability (**W**) are produced by specific shielded plasma oxidation treatments on PDMS substrates, as published before [20]. Each treatment will be discussed in detail below.

#### 4.1.1 PDMS preparation

PDMS films were prepared using a Sylgard 184 elastomer kit (DowDuPont). Elastomer (184A) and curing agent (184B) were mixed to a ratio of 10:1. The mixture was stirred and exactly 18 g was poured into a square Petri dish (12 × 12 cm) and left to degas at room temperature. Finally, the PDMS is cured at 70 °C overnight. The PDMS films were cut into 3.5 × 3.5 cm squares.

#### 4.1.2 Unidirectional wrinkle gradient formation

The wrinkle gradient is formed according to previously published methods [[76], [77], [78]]. The square PDMS films were placed in a custom-made stretching device and stretched for 20 % of its original length. A right-angled triangular prism mask of 2.2 cm long and 2 cm wide with an angle of 30° was placed on top of the stretched sample. The samples were placed in a plasma oven (Diener Electronic, model Atto, Ebhausen, Germany) and treated with air plasma at 25 mTorr for 650 s at maximum power, which oxidizes the surface of the PDMS films. After release of the stress, a wrinkled topography gradient was formed on the surface. To create a stiff layer on the entire PDMS surface, the samples were post treated for 600 s at 150 mTorr. These PDMS wrinkle gradients were then used as a mold for imprinting into untreated PDMS by pouring a fresh mixture of elastomer/curing agent (10:1) on top of the molds, followed by curing at 70 °C overnight. The molds were removed and the remaining topography (**T**) gradient imprints were used for further experiments.

#### 4.1.3 Double linear stiffness-wettability gradient formation

A double linear Stiffness-Wettability (**S|W**) gradient is formed by partially covering PDMS films with a right-angled triangular prism mask of 2 × 2 cm with an angle of 30° and performing an air plasma treatment at 40 mTorr for 20 s at maximum power [79]. The treatment oxidizes the PDMS films, which makes the surface stiffer and more hydrophilic, increasing both parameters from the closed to the open side of the mask. To prepare the DOG of Topography-Stiffness|Wettability (**T-S|W**), this treatment was performed on **T** gradient imprints, orthogonally placed to the direction of the first gradient. To remove the wettability gradient and generate the DOG of Topography-Stiffness (**T-S**), the **T-S|W** sample was fully oxidized at 1000 mTorr for 20 s to render the surface fully hydrophilic without affecting the stiffness.

#### 4.1.4 Wettability linear gradient

The DOG of Topography-Wettability (**T-W**) was prepared by performing a mild plasma treatment at 1000 mTorr for 60 s on top of the **T** gradient imprints, orthogonally placed to the direction of the topography gradient, partially shielding the PDMS with the same prism mask as described above. This milder treatment affects only the wettability of the surface without affecting the stiffness.

#### 4.1.5 Stiffness linear gradient

To prepare a linear stiffness (**S**) gradient, the double linear **S|W** gradient as described above is prepared on flat, untreated PDMS films. The **S** gradient was isolated from the **S|W** gradient by placing the samples into a desiccator containing a vial with 50 μL trichloro(propyl)silane (98 %, Sigma-Aldrich). A vacuum was applied overnight to yield a hydrophobic monolayer on top of the PDMS surface. The samples were rinsed with 70 % ethanol to remove remaining contaminants. To prepare the DOG of Stiffness-Wettability (**S-W**), a second treatment with the same prism mask as described above was performed at 1000 mTorr for 25 s orthogonally to the stiffness gradient.

#### 4.1.6 Surface characterization

The surface stiffness was determined with the use of a Nanoscope V Dimension 3100 microscope (Bruker, Billerica, MA, USA) with NanoScope Analysis software. All the data was obtained with the use of Bruker RTESPA-150 cantilevers (Veeco). The spring constant (2–3 N m^−1^) was determined before each experiment by thermal tuning and calibration on a reference glass slide. Three force curves were obtained on six different points on the gradient. The surface Young's modulus was calculated using the Sneddon Model, assuming a conical top geometry (with a tip radius of 8 nm and tip half angle of 18°) and a sample Poisson ratio of 0.3.

The surface wrinkled topography was determined with the use of a Nanoscope V Dimension 3100 microscope (Bruker, Billerica, MA, USA) with NanoScope Analysis as software. Bruker DNP-10 cantilever “D” (spring constant 0.006 N m^−1^) was used in contact mode to analyze the topographical features on the surface. The wavelength and amplitude of the wrinkled surface were determined in Nanoscope Analysis and were measured at 11 points on the direction of the gradient, with the 11th position outside of the mask, yielding the biggest wrinkle size. Three measurements were performed per position.

The wettability of the samples was measured as water contact angle (WCA), using a custom-built tensiometer, where 5 μL drops of Milli-Q water were placed on the surface using the sessile drop method. The WCA was measured on flat gradients, and not in combination with wrinkled topography. The wettability was measured immediately after plasma treatment on 5 locations along the gradient, with 3 measurements per position.

The gradient ranges of the values of the parameters topography, stiffness, and wettability for all four DOGs are shown in Table 1. Graphs of the different gradients can be found in Figure S1-2.

**Table 1:** Exact values of the DOG surface parameters.

Double Orthogonal Gradient	Topography	Stiffness DMT Modulus (MPa)	Wettability WCA (°)
	Wavelength λ (μm)		
	Amplitude (A) (nm)		
**Stiffness-Wettability (S**–**W)**	Flat	12.6 ± 5.7–478.9 ± 31.5	76.9 ± 6.9–19.3 ± 5.6
**Topography-Stiffness (T**–**S)**	λ: 1.3 ± 0.03–9.9 ± 0.2	12.6 ± 5.7–478.9 ± 31.5	<10 (hydrophilic)
	A: 74.5 ± 36.0–1024 ± 134		
**Topography-Wettability (T**–**W)**	λ: 1.3 ± 0.03–9.9 ± 0.2	13.0 ± 3.1–23.9 ± 2.3	92.7 ± 1.8–10.5 ± 1.7
	A: 74.5 ± 36.0–1024 ± 134		
**Topography-Stiffness|Wettability (T-S|W)**	λ: 1.3 ± 0.03–9.9 ± 0.2	12.6 ± 5.7–478.9 ± 31.5	85.3 ± 0.8–10.9 ± 2.1
	A: 74.5 ± 36.0–1024 ± 134

**Table 2:** Region of interest (ROI) material properties and translational plasma treatment for stiffness and wettability on topography imprints.

ROI	Screening				Translation	
	DOG	Topography Wavelength λ (μm)	Stiffness DMT Modulus (MPa)	Wettability WCA (°)	Treatment T	Treatment S/W
**Pos1**	**T-S|W**	10.0	47	82	25 mTorr 10 min 20 % stretch	40 mTorr 1s
**Pos2**	**T-S|W**	1.2	13	85	50 mTorr 10 s 30 % stretch	1000 mTorr 11s
**Pos3**	**T-S|W**	3.8	63	68	25 mTorr 60 s 30 % stretch	40 mTorr 2s
**Neg1**	**T-W**	4.8	16	91	25 mTorr 2.5 min 30 % stretch	1000 mTorr 8s
**Neg2**	**T-W**	1.4	16	92	50 mTorr 15 s 30 % stretch	1000 mTorr 8s
**Neg3**	**T-W**	7.6	16	92	25 mTorr 4 min 30 % stretch	1000 mTorr 8s

### 4.2 Cell culture of normal breast epithelial cells

Normal breast epithelial cells (MCF10a, Aldrich) were maintained in MCF10a Complete Medium (EP-ML-0525, Elabscience) containing 5 % horse serum, 20 ng mL^−1^ epidermal growth factor (EGF), 0.5 μg/mL hydrocortisone, 10 μg/mL insulin, 1 % non-essential amino acid solution (NEAA), and 1 % penicillin/streptomycin. Cell cultures were maintained at 37 °C and 5 % CO_2_.

### 4.3 Cell culture of breast cancer cells

Breast cancer cells (MCF7, Aldrich) were maintained in High Glucose Dulbecco's Modified Eagle Medium (DMEM, Thermo) supplemented with 10 % fetal bovine serum (FBS, Gibco, Thermo) and 1 % penicillin/streptomycin. Cell cultures were maintained at 37 °C and 5 % CO_2_.

### 4.4 Cell culture on DOG samples

Wettability gradients were prepared directly before proceeding to cell culture and stored in Milli-Q. This was done to remove the air-hydrophilic interface and maintain the surface properties in a polar environment, in order to avoid hydrophobic recovery, as previously characterized [36]. The DOG samples were cut into circular pieces of ⌀34 mm to fit into the well of a 6-wells plate. The edges of the DOG area were marked with a small cut to allow identification of its location during microscopy. Samples were sterilized with 70 % ethanol for 10 min and washed with sterile phosphate-buffered saline (PBS), twice, for 5 min. The samples were stored in PBS until cell seeding. Cells (MCF7 or MCF10a) were harvested with trypsin and seeded at a density of 5000 cells cm^−2^ directly on the DOG samples or on a tissue culture polystyrene (TCP) control and incubated at 37 °C and 5 % CO_2_. After 24 h or 72 h, the cells were washed with PBS, fixated with 4 % formaldehyde (VWR Chemicals) at room temperature for 20 min, and washed and stored in PBS at 4 °C until staining and imaging.

### 4.5 Immunofluorescence

Immunofluorescence staining was performed at room temperature. First, cells were permeabilized with 0.5 % Triton X-100 (Sigma-Aldrich/Merck) in PBS. Non-specific binding sites were blocked with 5 % bovine serum albumin (BSA, Sigma-Aldrich/Merck) in PBS for 30 min. Cells were incubated with primary antibody solution of Ki-67 (Abcam, ab15590, 1:500) in 1 % BSA in PBS for 1 h. Cells were washed three times with 1 % BSA in PBS, followed by secondary antibody staining with donkey anti-rabbit Alexa Fluor 647 (Jackson Immuno Research, 1:500), TRITC-labelled phalloidin (Sigma, P5282, 2 μg/mL), and DAPI (2 μg/mL) in 1 % BSA in PBS for 1 h. Cells were washed twice with 1 % BSA in PBS, once with PBS, and stored at 4 °C in the dark until imaging.

### 4.6 High-throughput fluorescence imaging and analysis

The cells were imaged using and automatically scanning Zeiss AxioObserver.Z1 TissueFAXs microscope (TissueGnostics, Vienna, Austria). The complete 6-well plate area was scanned and combined with TissueGnostics software, after which it was possible to identify the exact DOG location inside each well. For the TCP control and homogeneous sample ROI experiments, the analysis was performed in the same way, using three independent experiments.

**Tissuequest:** Image analysis of the cell area (phalloidin) and cell number (DAPI) was performed using TissueQuest analysis software. Cell number (nuclei count mm^−2^) was determined by a count of DAPI-positive nuclei, while the cell area was determined by measuring the area (μm^2^ per cell) in the phalloidin channel, normalized per cell. Data point areas of 2 by 2 mm (4 mm^2^) were drawn along the gradients, with 7 by 7 datapoint for the stiffness and topography gradients and 5 datapoints for the wettability gradients. This resulted in the following number of datapoints of each 4 mm^2^ per DOG: S-W: 35, T-S: 49, T-W: 35, T-S|W: 49. The material properties were measured at specific locations as described in the methods above, which were used to generate a trendline. The cell experiments data points’ exact locations were then fitted to the trendline to calculate the corresponding underlying material properties.

**ImageJ:** Image analysis of the percentage of proliferating cells (measured as percentage of Ki-67-positive nuclei) was performed using a custom-made ImageJ macro analyzing all images located in the 2 × 2 cm DOG area. This results in 12 x 17 images of 1741 × 1298 μm (2.26 mm^2^ data point area). In brief, the nuclei were determined using an overall background subtraction, thresholding, and particle analysis. A selection was created from the nuclei, and pasted over the Ki-67 channel (after background subtraction) followed by clearing the signal located outside the nuclei mask. The number of Ki-67 positive cells was determined and calculated as a percentage over the total number of cells. Early cluster formation density was calculated with the particle analyzer tool, segmenting objects in the phalloidin channel larger than 2000 μm^2^, corresponding to 2 or more cells clumped together. Single cells were quantified as objects smaller than 2000 μm^2^. Heatmaps and scatterplots of the results were generated using OriginPro 8.5.

Cell doubling time (in h) was calculated using the following formula: doubling time = [ (T_e_ – T_b_) x ln(2)]/[ ln(X_e_/X_b_)], where T_e_ is the end time in h, T_b_ is the begin time in h, X_e_ is the cell density at the end time, and X_b_ is the cell density at the begin time. This was calculated using the cell densities at 72h and 24h.

### 4.7 Statistical analysis

Data are shown as mean ± standard deviation, unless otherwise indicated. Statistical analyses were performed in GraphPad Prism 8.0.1 and Excel (Microsoft) was used for nonlinear regression. For comparison of groups, non-parametric Kolmogorov-Smirnov tests were performed. P < 0.05 was considered as significant. Partial least squares regression was performed on the data of Ki-67 after 72 h for MCF10a and MCF7 cells for each individual DOGs using three dependent variables (topography, stiffness, and wettability). Each variable was scaled by dividing by its standard deviation. For the T-S, T-W, and S-W DOG, which have one fixed parameter, an offset term was added to avoid numerical errors caused by a standard deviation of zero. Leave-one-out (LOO) cross-validation was used to determine the number of components. Plots were created with the R package “ggplot2” version 3.4.2.

### 4.8 Translational cell experiments

Regions of interest (ROIs) were translated towards homogeneous substrates using treatments listed in Table 2. First, PDMS topography molds were prepared and imprinted into fresh 10:1 as described above, followed by a treatment to alter the stiffness and wettability. Samples of each ROI were 1.9 cm^2^, to fit into the well of a 24-well plate. Cell culture studies were repeated for these ROIs as described before. Cells were seeded at a higher seeding density, 5000 cells cm^−2^, and lower seeding density, 1000 cells cm^−2^, respectively.

## CRediT authorship contribution statement

**Lisa E. Tromp:** Writing – original draft, Visualization, Methodology, Investigation, Formal analysis, Conceptualization. **Rik de Jong:** Writing – review & editing, Investigation. **Torben A.B. van der Boon:** Writing – review & editing, Methodology. **Alejandro Reina Mahecha:** Software. **Ruud Bank:** Writing – review & editing, Conceptualization. **Jan de Boer:** Writing – review & editing, Conceptualization. **Patrick van Rijn:** Writing – review & editing, Supervision, Conceptualization.

## Data availability statement

The data that support the findings of this study are available from the corresponding authors upon reasonable request.

## Ethics approval and consent to participate

Not applicable.

## Declaration of competing interest

P.v.R. is also co-founder, scientific advisor, and share-holder of BiomACS BV, a biomedical oriented screening company.
