# Supplementary material for: Harnessing the power of physicochemical material property screening to direct breast epithelial and breast cancer cells
Source: Bioact Mater. 2025 Apr 26;50:494–509. doi: 10.1016/j.bioactmat.2025.04.003 (PMC12059224; doi:10.1016/j.bioactmat.2025.04.003)
Supplement: Multimedia component 1 [file mmc1.docx]

# Harnessing the Power of Physicochemical Material Property Screening to Direct Breast Epithelial and Breast Cancer Cells

## Supplementary Figures

**Figure S1** **Surface topography gradient characterization**. **a**, the wavelength and amplitude of the wrinkles are increasing from the closed to the open side of the mask during plasma treatment, and **b**, shows the quantified increase in wrinkle size.

**Figure S2** **Sequential plasma treatments lead to different stiffness-wettability gradients.** **a**, The Stiffness-Wettability Linear Gradient is generated by the coupled plasma treatment. **b**, Silanization of **a** leads to a fully hydrophobic surface and generates an isolated Stiffness Linear Gradient. **c**, Adding a second uncoupled treatment to **b** leads to the final Stiffness-Wettability DOG. **d**, The uncoupled treatment affects only the wettability and leads to an isolated Wettability Linear Gradient. **e**, Water contact angle images of the wettability gradient from the coupled plasma treatment.

**Figure S3a** **Overview of stitched fluorescence images of the entire DOG region.** Dashed white squares indicate each 20 x 20 mm DOG. Phalloidin is indicated in green, DAPI in blue, and Ki-67 in red. Scale bar is 5 mm.

**Figure S3b** **Overview of stitched fluorescence images of the entire DOG region: MCF10a cells – S-W DOG.** Dashed white squares indicate each 20 x 20 mm DOG, black scale bar is 5 mm. Phalloidin is indicated in green, DAPI in blue, and Ki-67 in red. In-set images white scale bar is 100 µm.

**Figure S3c** **Overview of stitched fluorescence images of the entire DOG region: MCF10a cells – T-S DOG.** Dashed white squares indicate each 20 x 20 mm DOG, black scale bar is 5 mm. Phalloidin is indicated in green, DAPI in blue, and Ki-67 in red. In-set images white scale bar is 100 µm.

**Figure S3d** **Overview of stitched fluorescence images of the entire DOG region: MCF10a cells – T-W DOG.** Dashed white squares indicate each 20 x 20 mm DOG, black scale bar is 5 mm. Phalloidin is indicated in green, DAPI in blue, and Ki-67 in red. In-set images white scale bar is 100 µm.

**Figure S3e** **Overview of stitched fluorescence images of the entire DOG region: MCF10a cells – T-SW DOG.** Dashed white squares indicate each 20 x 20 mm DOG, black scale bar is 5 mm. Phalloidin is indicated in green, DAPI in blue, and Ki-67 in red. In-set images white scale bar is 100 µm.

**Figure S3f** **Overview of stitched fluorescence images of the entire DOG region: MCF7 cells – S-W DOG.** Dashed white squares indicate each 20 x 20 mm DOG, black scale bar is 5 mm. Phalloidin is indicated in green, DAPI in blue, and Ki-67 in red. In-set images white scale bar is 100 µm.

**Figure S3g** **Overview of stitched fluorescence images of the entire DOG region: MCF7 cells – T-S DOG.** Dashed white squares indicate each 20 x 20 mm DOG, black scale bar is 5 mm. Phalloidin is indicated in green, DAPI in blue, and Ki-67 in red. In-set images white scale bar is 100 µm.

**Figure S3h** **Overview of stitched fluorescence images of the entire DOG region: MCF7 cells – T-W DOG.** Dashed white squares indicate each 20 x 20 mm DOG, black scale bar is 5 mm. Phalloidin is indicated in green, DAPI in blue, and Ki-67 in red. In-set images white scale bar is 100 µm.

**Figure S3i** **Overview of stitched fluorescence images of the entire DOG region: MCF7 cells – T-SW DOG.** Dashed white squares indicate each 20 x 20 mm DOG, black scale bar is 5 mm. Phalloidin is indicated in green, DAPI in blue, and Ki-67 in red. In-set images white scale bar is 100 µm.

**Figure S4 Scatter plots of cell proliferation for both 24 and 72 hours**. An extended version of **Figure 1b**, which shows both the 24 and 72 hours scatter plots that summarize all datapoints from the screening form low to high. Tissue culture polystyrene (TCP) was used as a control. The 10% lowest and 10% highest scoring datapoints are shown on the side and are shown to be significantly different (p<0.0001).

**Figure S5a Cell density screening results.** Heatmaps of cell density (average of n=3), quantified as the number of cells per mm^2^ on the different DOGs for the MCF10a and MCF7 cells after 24 and 72 hours.

**Figure S5b Cell density screening results.** Scatter plots of screening data that summarize all datapoints from low to high. Tissue culture polystyrene (TCP) was used as a control. The 10% lowest and 10% highest scoring datapoints are shown on the side and are shown to be significantly different (p<0.0001).

**Figure S6a Cell area screening results.** Heatmaps of cell area (average of n=3), quantified as area in µm^2^ per cell, on the different DOGs for the MCF10a and MCF7 cells after 24 and 72 hours.

**Figure S6b Cell area screening results.** Scatter plots of screening data that summarize all datapoints from low to high. Tissue culture polystyrene (TCP) was used as a control. The 10% lowest and 10% highest scoring datapoints are shown on the side and are shown to be significantly different (p<0.0001).

**Figure S7** **Scatter plots comparing the effect of each individual physicochemical parameter of the DOG between MCF10a and MCF7 cells.** The material parameters (topography, wettability, and stiffness on the same data points, shown with the corresponding color scale) were shown on the biological output (cell density and cell area) for the MCF10a vs. the MCF7 cells.

**Figure S8 Difference in proliferation (% Ki-67-positive cells) for flat and topographical surfaces**. All datapoints from the S-W DOG are grouped and show a statistically significant difference (p<0.0001) with all datapoints from the DOGs containing topography (T-S|W, T-S, T-W).

**Figure S9 Fluorescence images of both the MCF10a and MCF7 cells after 72 hours.** Images are shown for respective regions of interest (ROIs) shown in Figure S4a where the cell proliferation is higher for the MCF10a cells compared to the MCF7 cells. Scale bar is 200 μm.

**Figure S9, continued.**

**Figure S9, continued.**

**Figure S10 ROI characterization.** **a**, Topography characterization of wavelength (µm) and amplitude (nm) of each translated ROI. **b**, Stiffness and wettability characterization of plasma treatments used to create translated ROIs.

**Figure S11** Fluorescence images of MCF10a and MCF7 cells after 72 hours of translational ROI experiments with high seeding density (5000 cells cm^-2^). Cells are stained for Ki-67 (red), phalloidin (green), and DAPI (blue). Scale bar is 200 µm.

**Figure S12** Fluorescence images of MCF10a and MCF7 cells after 72 hours of translational ROI experiments with low seeding density (1000 cells cm-2). Cells are stained for Ki-67 (red), phalloidin (green), and DAPI (blue). Scale bar is 200 µm.

**Figure S13 Nonlinear regression analyses of individual material properties after 24 hours of cell culture.** Data points were plotted with the value for topography, wettability, or stiffness against cell proliferation, cell density, and cell area and nonlinear second-order polynomial regression was performed. Trend lines with the highest R^2^ values are shown on each graph, with values higher than 0.4 deemed as representative, shown in orange.

**Figure S14 Partial Least Squares (PLS) analysis**. The relative variable importance (in %) of the parameters stiffness (MPa), topography (µm), and wettability (WCA) for the assesses cell proliferation (Ki67 %) for both MCF10a and MCF7 cells. Positive values indicate a positive correlation, while negative values indicate a negative correlation. The total for each DOG is 100%.

**Figure S15** R-squared (R^2^) values and number of components used for the partial least squares regression. In each case, the highest number of components was used to describe the data.

**Figure S16 3d plots of Figure 8a.** Each axis represents a variable: cluster density is quantified as every individual phalloidin area in contact larger than 2000 μm^2^ mm^-2^, corresponding to two or more cells. Single cell density is quantified as every individual phalloidin area in contact smaller than 2000 μm^2^ mm^-2^, corresponding to single cells. The material properties are shown on the 3^rd^ axis.

**Figure S17 Cluster density of MCF7 cells after 72 hours of cell culture.** Cluster density is quantified as every individual phalloidin area in contact larger than 2000 μm^2^ mm^-2^, corresponding to two or more cells. **a**, Cluster density is plotted against average cluster area (μm^2^ per cell). Linear regression analysis was performed and the dotted orange line shows the trend line with R^2^ value. **b**, The underlying topography wrinkle size in μm was plotted against average cluster density. **c**, All datapoints of the cluster area (μm^2^/cluster) from the flat S-W DOG are grouped and show a statistically significant difference (p<0.0001) with all datapoints from the DOGs containing topography (T-S|W, T-S, T-W).
